# Functional status of pediatric patients with trauma and risk factors for mortality from a single center in China

**DOI:** 10.3389/fped.2023.1051759

**Published:** 2023-05-03

**Authors:** Yu-Hang Yang, Tie-Ning Zhang, Ni Yang, Wei Xu, Li-Jie Wang, Shan-Yan Gao, Chun-Feng Liu

**Affiliations:** ^1^ Department of Pediatrics, Shengjing Hospital of China Medical University, Shenyang, China; ^2^ Department of Clinical Epidemiology, Shengjing Hospital of China Medical University, Shenyang, China

**Keywords:** wounds and injuries, mortality, functional status, injury severity score, motor disorders

## Abstract

**Introduction:**

The influence of reduced functional status has become increasingly relevant because of the gradual decline in mortality rate over the recent years. Nonetheless, only a few studies investigating the functional status of patients with trauma at hospital discharge have been conducted. This study aimed to identify the risk factors influencing the mortality rate in pediatric trauma survivors at a pediatric intensive care unit and analyze their functional status using the Functional Status Scale (FSS).

**Methods:**

A retrospective analysis was conducted at Shengjing Hospital of China Medical University. Children admitted to the pediatric intensive care unit between January 2015 and January 2020 who met the trauma diagnostic criteria were included. The FSS score and the Injury Severity Score (ISS) were recorded upon admission and discharge, respectively. Clinical data were compared between the survival and non-survival groups to identify the risk factors for poor prognosis. The risk factors for mortality were identified using multivariate and univariate analyses.

**Results:**

A total of 246 children {59.8%, male; median [interquartile range (IQR)] age: 3 [1–7] years} were diagnosed with trauma (including head trauma, chest trauma, abdominal trauma, and extremity trauma). Of these patients, 207 were discharged, 11 dropped out mid-treatment, and 39 died (hospital mortality rate, 15.9%). Upon admission, the median FSS and trauma scores were 14 (IQR, 11–18) and 22 (IQR, 14–33) points, respectively. At discharge, the FSS score was 8 (IQR, 6–10) points. The patient clinical status improved with a ΔFSS score of −4 (IQR, −7, 0) points. At hospital discharge, 119 (48.3%), 47 (19.1%), 27 (11.0%), 12 (4.8%), and 2 (0.9%) survivors had good, mildly abnormal, moderately abnormal, severely abnormal, and very severely abnormal function, respectively. Reduced functional status in patients was categorized as follows: motor, 46.4%; feeding, 26.1%; sensory, 23.2%; mental, 18.4%; and communication, 17.9%. In the univariate analysis, ISS >25 points, shock, respiratory failure, and coma were independently associated with the mortality rate. Multivariate analysis revealed that the ISS was an independent risk factor for mortality.

**Conclusion:**

The mortality rate of patients with trauma was high. ISS was an independent risk factor for mortality. Mildly reduced functional status remained at discharge and was reported in nearly half of the discharged patients. Motor and feeding functions were the most severely impacted domains.

## 1. Introduction

Trauma has been reported as the leading cause of death among children and adolescents, accounting for 20% of deaths (1). Over the last decade, the proportion of patients with trauma has increased, accounting for 16.3%–18.1% of hospitalizations (2). Despite the increased severity and morbidity of injuries among hospitalized patients with trauma, a decline in mortality rate has been observed (3). However, as the number of trauma survivors increase, it is increasingly recognized that many of these patients suffer from reduced functional status following discharge from the pediatric intensive care unit (PICU). Moreover, pediatric patients with trauma often exhibit several behavioral abnormalities, such as psychiatric disorders, feeding difficulties, and post-traumatic stress disorder (4–6). Recent studies have reported that reduced functional status is common among pediatric patients with trauma. Currently, the overall trauma rate is 17.3% (7). One study reported that approximately 65% of patients with trauma had an Abbreviated Injury Scale (AIS) score of ≥3 points and that reduced functional status at discharge was present in 16% of the patients, increasing to 43% among patients with traumatic brain injury (8). Among survivors, the quality of life was considerably poor in patients with an abnormal discharge Functional Status Scale (FSS) score at the 6-month follow-up, particularly in older patients and in those with penetrating type injury, severe brain injury, and spinal injury (9). Therefore, understanding the mortality rate and functional status of pediatric patients with trauma is crucial.

A study from US trauma centers showed that the mortality rate of injured children receiving initial care is relatively low at 1.5% (10). Therefore, it is not enough to focus on improvements in mortality in patients with trauma. We also need to focus on what concerns remain when patients are discharged from the hospital. Currently, the tools used to evaluate pediatric trauma include the Injury Severity Score (ISS), the New ISS (NISS), and the Pediatric Trauma Score (PTS). However, the ISS and NISS are currently regarded as predictors for mortality, and the PTS has limited effectiveness in determining the functional status of pediatric patients with trauma because it is not universally applicable (11, 12). The FSS is an age-independent assessment of pediatric functional status used in a wide range of studies (13). This instrument has been reported to provide an objective, age-independent measure of reduced functional status (14). Nonetheless, while the FSS adequately evaluates the prognosis of patients with a single injury, such as traumatic brain injury, the research on its role in the assessment of prognosis in patients with multiple traumas is limited.

Therefore, this retrospective study of clinical data of pediatric patients with trauma was conducted to analyze the mortality rate and functional status of pediatric trauma survivors and identify the risk factors influencing the mortality rate.

## 2. Methods

### 2.1. Study population

This study included all children with trauma who were admitted to the PICU at Shengjing Hospital of China Medical University between January 2015 and January 2020. We searched the Hospital Information System (HIS) database, which is a medical record database for all admissions and includes diagnostic and clinical codes representing “Principal diagnosis” and “All listed diagnoses” for trauma (ICD-10 code T14. 90XA) or polytrauma (ICD-10 code T07) cases. Patient medical records were examined to confirm the diagnoses. The inclusion criteria for this study were as follows: age between 1 month and 14 years and diagnosis of trauma or polytrauma. The following exclusion criteria were applied: presence of missing data and PICU hospitalization for <24 h.

### 2.2. Ethics statement

The study was approved by the Institute Research Medical Ethics Committee of Shengjing Hospital (2022PS689K). The requirement for the acquisition of informed consent was waived owing to the retrospective nature of this study.

### 2.3. Data collection

The data collection approach involved a retrospective analysis of patient medical records from the HIS database to collect and analyze FSS of patients at the time of admission and discharge. Specifically, the researchers collected data on FSS by reviewing the patients' medical records. Trained research coordinators collected data from available medical records and discussed the data with clinicians when problems arose. A retrospective analysis of clinical and demographic data was conducted using the HIS database. The admission FSS score was determined by trained research coordinators who conducted an assessment of the patient's functional abilities at the time of hospital admission based on the record in HIS database. Clinical and functional outcomes upon both admission and discharge were assessed using the FSS, and the difference in the FSS scores between discharge and admission was calculated. Demographic data (age and sex), mortality rate, FSS score at admission, and FSS score at discharge obtained from electronic medical records were retrospectively collected and analyzed. The following trauma features were documented: mechanism and type of trauma, location of trauma, and the time of medical intervention; prehospital cardiopulmonary resuscitation; respiratory failure; convulsions; and shock.

### 2.4. Definitions

In-hospital mortality was defined as death occurring in the hospital. Dropped out mid-treatment indicated that an individual withdrew from a medical or therapeutic treatment before completing the full course of the treatment. The FSS was used to assess the survivor's functional status. It includes six domains (mental status, sensory, communication, motor, feeding, and respiratory). The domain scores range from 1 (normal) to 5 (extreme dysfunction) points. Scores range from 6 to 30 points, with 6–7, 8–9, 10–15, and 16–21 points indicating a normal, mildly dysfunctional, moderately dysfunctional, and seriously dysfunctional condition, respectively (15). Severe craniocerebral trauma was defined as craniocerebral trauma with a Glasgow Coma Scale score of ≤8 points. Upon admission, all patients were evaluated using the AIS. Based on the patients' survival status at discharge, the pediatric patients with trauma were divided into survival and non-survival groups. The AIS ranks the severity of an injury on a 6-point scale (16) according to the following six body regions: (1) head and neck, (2) face, (3) chest, (4) abdomen, (5) extremities and pelvic girdle, and (6) external injuries. The ISS was calculated by squaring the three highest AIS scores. Severe trauma was defined as an ISS of >25 points (17).

### 2.5. Statistical analyses

Continuous variables are presented as medians [interquartile ranges (IQRs)], whereas categorical variables are expressed as counts (percentages). Logistic regression analyses were conducted to compare trauma outcomes and patient characteristics. Variables with a *P*-value of <0.2 in the univariate logistic regression analysis were included in a subsequent multivariate logistic regression analysis. All statistical analyses were performed using Stata version 13.0 (StataCorp, College Station, TX, USA). Statistical significance was defined as a two-tailed *P*-value of <0.05.

## 3. Results

### 3.1. Study population

A total of 258 pediatric patients met the inclusion criteria; however, 12 patients were subsequently excluded from the study due to missing data (*n* = 7) and admission within <24 h (*n* = 5) (Supplementary Figure S1). Overall, 246 patients [59.8%, male; median (IQR) age: 3 (1–7) years] were diagnosed with trauma (including head trauma, chest trauma, abdominal trauma, and extremity trauma). Of these, 207 patients (84.1%) survived to discharge, whereas 39 patients (15.9%) died during hospitalization. Further, among the surviving patients, 11 patients (4.4%) discontinued treatment. Vehicle accidents and falls accounted for 51.6% and 44.7% of all common etiologies of trauma, respectively, with 203 (82.5%) cases of cranial trauma and 67 (27.2%) cases of severe cranial trauma. Among patients aged ≤3 years, 57% and 40.4% were hospitalized for fall and car accident injuries, respectively. In patients aged >3 years, 61.4% and 34.1% were hospitalized for car accident and fall injuries, respectively. The baseline characteristics of the participants are presented in Table 1.

**Table 1:** Baseline characteristics.

Variable	Total^a^
**Age group (years)**	
1–4	149 (60.6)
5–9	70 (28.5)
10–14	27 (11.0)
Sex (male)	147 (59.8)
Total hospital length of stay (days)	10 (6–16)
**Body regions with an AIS** ^**b**^	
1	104 (42.3)
2	138 (56.1)
≥3	4 (1.6)
**ISS^c^**	
1–8	10 (4.1)
9–15	57 (23.2)
16–25	80 (32.5)
>25	99 (40.2)
Survival rate (%)	207 (84.2)
Change in FSS among survivors	5 (3–8)
**Injury mechanism**	
Fall	110 (44.7)
Transport, other or motorcycle	127 (51.6)
Other	9 (3.7)
Shock (yes)	36 (14.6)
Respiratory failure (yes)	69 (28.1)
Coma (yes)	112 (45.5)
Convulsions (yes)	42 (17.1)
Secondary infection (yes)	68 (27.6)
Craniocerebral injury (yes)	203 (82.5)

AIS, abbreviated injury scale; FSS, functional status scale; ISS, injury severity score; IQR, interquartile range.

^a^ Categorical variables are presented as counts (percentages), whereas continuous variables are presented as median (IQR).

^b^ AIS scores range from 1 to 6 points, with higher scores indicating a more severe injury. A score of ≥3 points indicates serious, severe, or critical injury.

^c^ ISSs range from 1 to 75 points, with higher scores indicating a more severe injury. A score of ≥24 points indicates serious, severe, or critical injury.

### 3.2. Change in the FSS score from baseline

**Figure 1:**
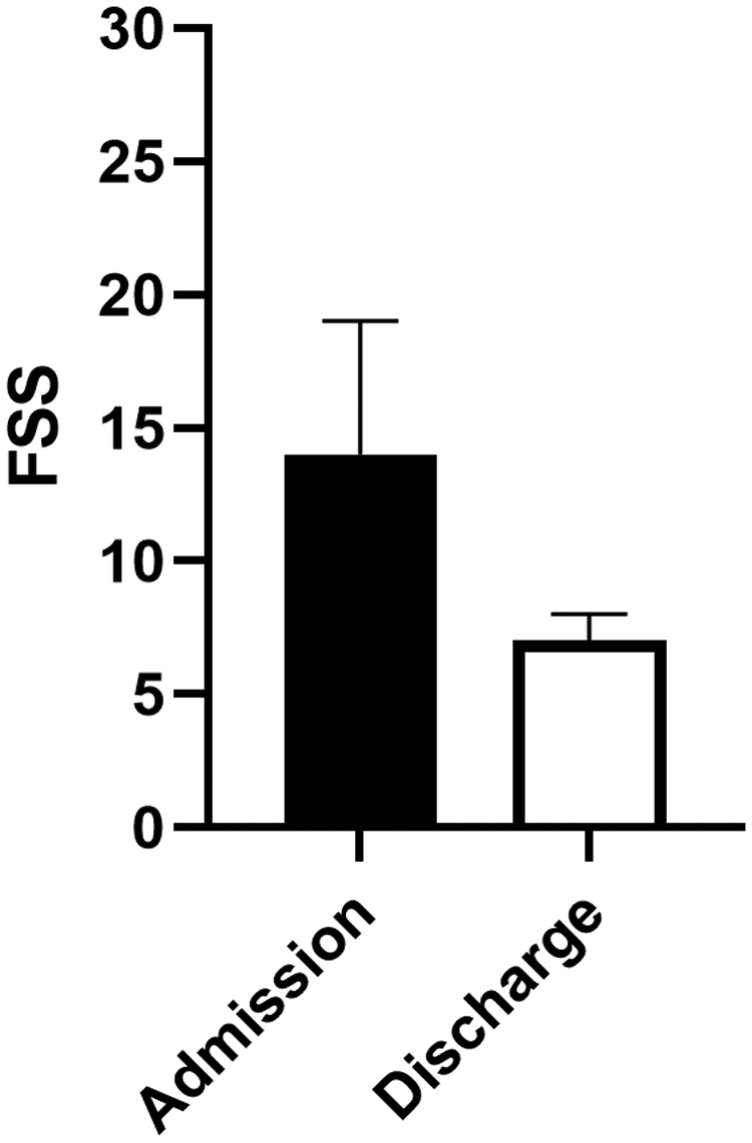
Change in the functional status score from baseline to discharge among survivors.

Patients had a better FSS score at discharge than that at baseline, with an ΔFSS score of −4 (−7, 0) points. The change in the median FSS score among trauma survivors is presented in Figure 1. The median FSS score was 14 (IQR, 11–18) points upon admission and 8 (IQR, 6–10) points at discharge, whereas the median ISS was 22 (IQR, 14–33) points. A total of 207 patients (84.1%) survived to discharge. Upon hospital discharge, 119 (48.3%) survivors presented with good function, 47 (19.1%) with mildly abnormal function, 27 (11.0%) with moderately abnormal function, 12 (4.8%) with severely abnormal function, and 2 (0.9%) with very severely abnormal function.

### 3.3. FSS domain of discharge

**Figure 2:**
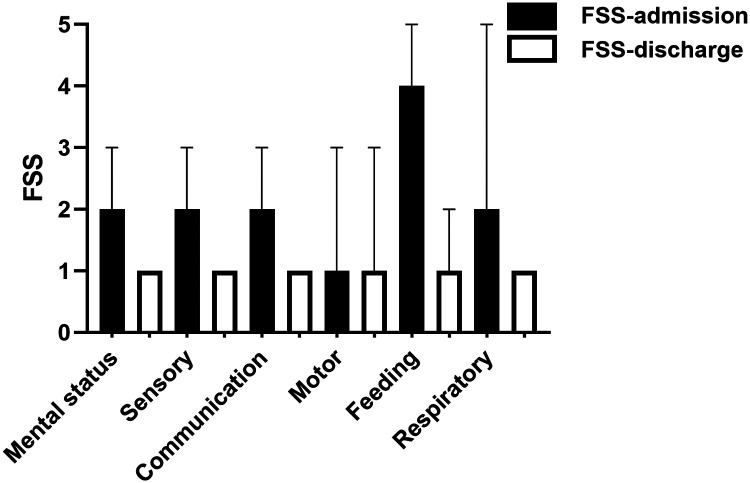
Functional status score domains at baseline and discharge among survivors.

**Figure 3:**
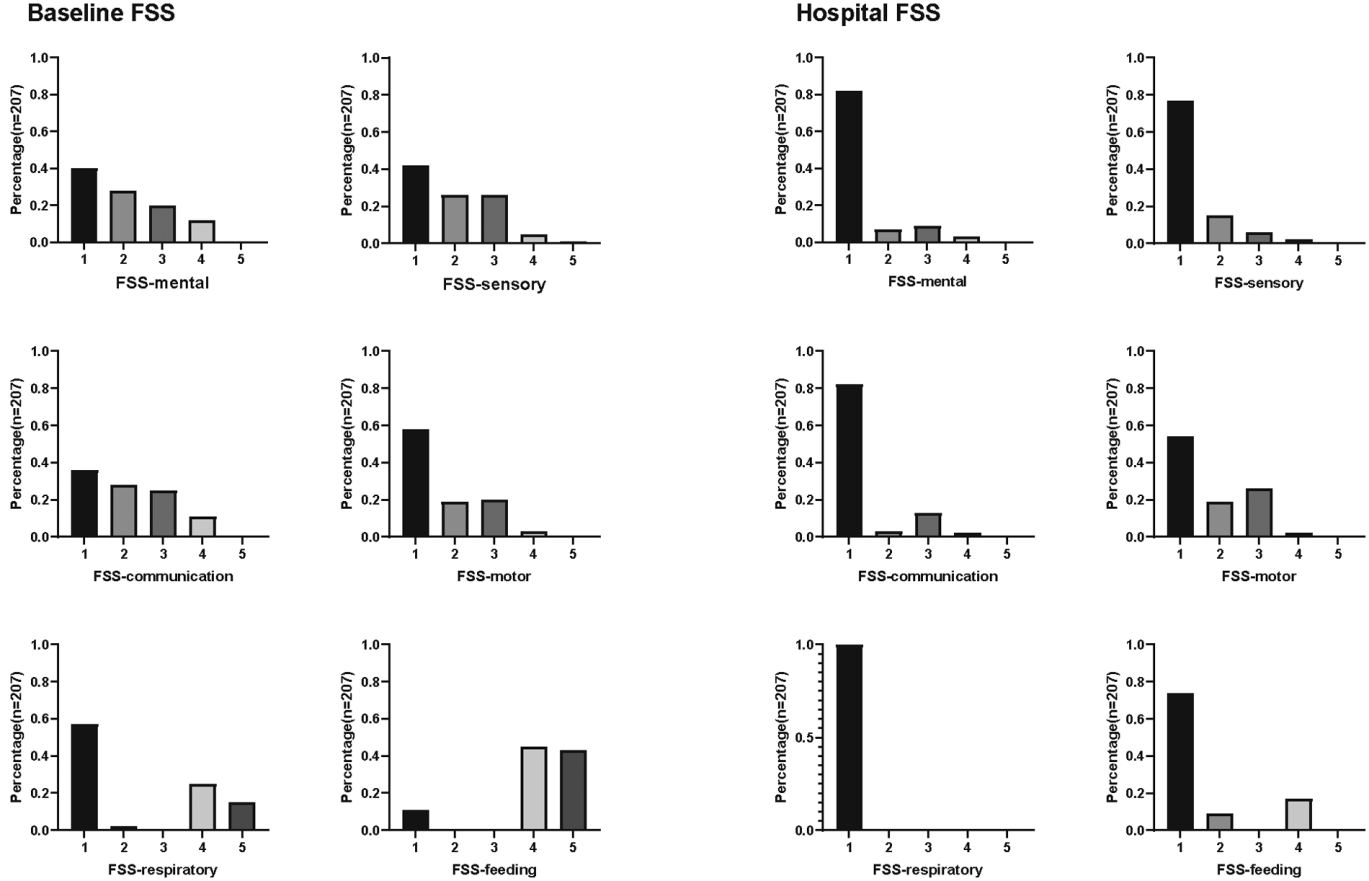
Histograms of FSS domain scores at baseline and discharge among survivors.

Upon admission, the feeding function was severely abnormal, whereas mental, sensory, communication, and respiratory functions were mildly abnormal. In contrast, the motor function was normal (Figure 2). At discharge, all functional scores were within the normal range. We analyzed the percentage of each functional score at discharge. Upon discharge, patients exhibited some degree of functional impairment in most domains, particularly in the motor and feeding domains (Figure 3). The proportion of patients with reduced respiratory functional status at discharge was the lowest among the domains assessed. Among survivors, 46.4%, 26.1%, 23.2%, 18.4%, and 17.9% showed reduced motor, feeding, sensory, mental, and communication functional status, respectively. Among patients with movement disorders, 19.3% had functional impairment in one limb and 25.6% had functional impairment in two or more limbs. Functional abnormalities were mainly associated with fractures, soft tissue injuries, brachial plexus injuries, venous thrombosis of the lower extremities, and treatment factors, such as bandages and casts. Severe motor function abnormalities were rare, with only 2.4% of patients demonstrating significantly reduced motor functional status, such as loss of voluntary motor function or poor head control. In the feeding domain, 9.2% of patients required special formulas (i.e., hydrolyzed infant formulas, amino acid-based formula, and preterm milk), and in 16.9% of the patients, swallowing function did not return to normal levels and a feeding tube was required. No patients required total parental nutrition at discharge.

### 3.4. Statistical analysis results

Univariate analysis revealed that an ISS of >25 points, shock, respiratory failure, and coma were independently associated with the mortality rate. Furthermore, the non-survivor group had a higher percentage of patients with an ISS of >25 points, shock, respiratory failure, severe craniocerebral trauma, and coma. Age, sex, proportion of convulsions, body regions with an AIS, injury mechanism, and secondary infection did not differ between the two groups (Table 2). Multivariate analysis indicated that ISS was independently associated with mortality in patients with trauma (Table 3).

**Table 2:** Baseline characteristics of survivors and non-survivors after injury.

Variable	Non-survivors^a^ (*n* = 39)	Survivors^a^ (*n* = 207)	*P*-value
Age group (years)			0.27
1–4	20 (51.3)	129 62.3)	
5–9	14 (35.9)	56 (27.1)	
10–14	5 (12.8)	22 (10.6)	
Sex (male)	25 (64.1)	122 (58.9)	0.55
Total hospital length of stay (days)	2 (1–4)	12 (9–17)	<0.001
Body regions with an AIS^b^			0.96
1	16 (41.0)	88 (42,5)	
2	23 (59.0)	115 (55.6)	
≥3	0 (0.0)	4 (1.9)	
ISS^c^			<0.001
1–8	0 (0.0)	10 (4.8)	
9–15	2 (5.1)	55 (26.6)	
16–25	4 (10.3)	76 (36.7)	
>25	33 (84.6)	66 (31.9)	
Injury mechanism			0.21
Fall	16 (41.0)	94 (45.4)	
Transport, other or motorcycle	19 (48.7)	108 (52.2)	
Other	4 (10.3)	5 (2.4)	
Shock (yes)	17 (43.6)	19 (9.2)	<0.001
Respiratory failure (yes)	36 (92.3)	33 (15.9)	<0.001
Coma (yes)	33 (84.6)	79 (38.2)	<0.001
Convulsions (yes)	6 (15.4)	36 (17.4)	0.76
Secondary infection (yes)	3 (7.7)	65 (31.4)	0.006
Craniocerebral injury (yes)	34 (87.2)	169 (81.6)	0.41

AIS, abbreviated injury scale; FSS, functional status scale; ISS, injury severity score; IQR, interquartile range.

^a^ Categorical variables are presented as counts (percentages), whereas continuous variables are presented as median (IQR).

^b^ AIS scores range from 1 to 6 points, with higher scores indicating a more severe injury. A score of ≥3 points indicates serious, severe, or critical injury.

^c^ ISSs range from 1 to 75, with higher scores indicating a more severe injury. A score of ≥24 points indicates serious, severe, or critical injury.

**Table 3:** Association between the ISS and survival rate.

	OR (95% CI)	*P*-value
**ISS**		
Crude model	5.63 (2.73–11.60)	<0.001
Adjusted model 1^a^	5.90 (2.81–12.38)	<0.001
Adjusted model 2^b^	8.81 (3.14–24.75)	<0.001

ISS, injury severity score.

^a^ Adjusted for age and sex.

^b^ Adjusted for variables in model 1 and total hospital length of stay.

## 4. Discussion

In this study, the mortality rate of patients with trauma was 15.9%. ISS was identified as an independent risk factor for mortality rate. Mildly reduced functional status remained at discharge and was reported in nearly half of the discharged patients. Motor and feeding functions were the most severely impacted domains.

The patients in this study had a higher mortality rate than those in other pediatric trauma studies (10). The high mortality rate in our center could be attributed to the following reasons. First, our center is a national regional pediatric medical center in Northeast China. Thus, critically ill patients, including those from the northeastern provinces and Inner Mongolia, are transferred to our PICU. In our study, the median ISS upon admission was 22 points, and the mortality rate was 15.9%. A previous study has reported that among patients with severe trauma, the median ISS was 22 points, with a mortality rate of 17.5%, which is consistent with that reported in our study (17). Second, our hospital has a trauma center and admits numerous pediatric patients with trauma, but only critically ill patients are transferred to the PICU for treatment, while other patients are admitted in general wards, such as the thoracic surgery and neurosurgery wards. Thus, the patients included in this study were all patients in critical condition. Third, because of the extremely poor prognosis and/or financial constraints, some patients (4.4%) did not complete all treatments. They stopped treatment in the middle of the process, which increased the mortality rate, despite some patients having a chance of survival had they continued with their treatment.

The functional status at discharge is a major clinical concern. In this study, most patients survived. Although the overall functional status at discharge was significantly improved compared with that at admission, mild functional impairment remained in nearly half of the patients. A previous study concluded that although reduced functional status was relatively low (approximately 9%) in pediatric patients with trauma, a substantial proportion of patients had functional impairment upon hospital discharge and returned to normal function within 6 months of discharge (18). At discharge, functional impairments in cognitive, motor, and feeding functions were observed, and the lowest prevalence of reduced functional status was reported in the respiratory domain. Additionally, 46.4% of patients had reduced motor functional status due to limb fractures, spinal cord injury, thrombosis, peripheral nerve injuries, dystonia, and plaster cast immobilization. Compared with other functions, the motor function was similar at both admission and discharge, despite the fact that the functional status was worse at discharge. The proportion of patients with ≥2 limb movement disorders (motor FSS score, 3 points) increased compared with that at admission, suggesting that motor function may require a longer rehabilitation time. Further follow-up examinations are essential to evaluate motor function recovery.

Upon admission, 28.1% of patients had acute respiratory failure caused by direct thoracic injury (including lung contusion, pleural effusion, rib fracture, and pleural effusion), deep coma, hemorrhagic shock, cardiopulmonary resuscitation, and following emergency surgery. Reduced functional status in the respiratory domain is reportedly low among patients with trauma (7). Nevertheless, the respiratory function recovered relatively rapidly after injury among survivors at discharge. One study revealed that in most cases involving patients with trauma, the respiratory FSS score was normal upon discharge, whereas a small proportion of patients had an FSS score of 3 points and still required oxygen (19). Reportedly, approximately 42% of patients with trauma required mechanical ventilation for an average of 3 days and an average length of ICU hospitalization of 7.5 days, indicating a short recovery time (20). Another study reported that most patients with lung contusion have a good prognosis and recover within 5–7 days of admission (21). Our findings are consistent with these data.

One of the most prevalent problems influencing long-term quality of life is feeding disorder. Reportedly, 9.1% and 47.9% of patients in the PICU exhibited a 3- and 2-point increase in the feeding domain, respectively (14). A post-discharge study revealed that 12.1% of patients still had feeding difficulties at 2 months following discharge from the PICU (22). Feeding-related reduced functional status has been reported to occur frequently among patients with craniocerebral, abdominal, and thoracic traumas (7). However, clinical data on feeding disorders in pediatric patients with trauma are insufficient. Approximately 26.1% of survivors had reduced feeding functional status at discharge, and most patients were discharged on nasogastric tube feeding. Other patients with feeding disorders required preterm milk, special formulas, liquid meals, and feeding assistance. Nevertheless, no patients required total parenteral nutrition at discharge. Thus, feeding disorders may be common among patients in the PICU. The most commonly identified reasons for feeding disorders are endotracheal intubation, neuromuscular weakness, and delirium or disruption of consciousness (23). Our study revealed that feeding disorders might be attributed to the fact that the brain function did not recover after discharge and that patients remained unconscious and were unable to eat or drink independently. The proportion of patients with feeding disorders caused by abdominal injury was small. Following hospital discharge, patients should continue to receive long-term rehabilitation therapy and follow-up care.

### 4.1. Limitations

The study has some limitations. First, this was a retrospective single-institution study with a limited sample size. Therefore, there is a risk of missing data and information bias. Second, the research results had limited generalizability. This study did not include patients hospitalized in pediatric surgical wards; cardiac and thoracic surgical wards; ear, nose, and throat surgical wards; or general surgery wards. Thus, whether these risk factors apply to other hospital settings, particularly those outside ICUs, remains unclear. Third, the FSS does not provide extensive clinical assessments for attention-deficit hyperactivity disorder/attention-deficit disorder, subcutaneous emphysema, lung contusion, organ injuries (abnormal liver function test or abnormal renal function), and incontinence. Furthermore, 4.4% of patients discontinued treatment, indicating that the reduced functional status rate may have been overestimated as it included discharged patients who had discontinued treatment. Moreover, the effect of treatment factors on the patients' prognosis was not evaluated in our study, which would have influenced our conclusion. Finally, the post-discharge functional status was not assessed at follow-up outpatient clinic visits. Future research should focus on the functional status of pediatric patients with trauma, and multicenter, long-term clinical studies are required to generate robust data.

### 4.2. Conclusion

In this study, the mortality rate of patients with trauma was high. Trauma severity affected the mortality rate. Mildly reduced functional status remained at discharge and was reported in nearly half of the discharged patients. Motor and feeding functions were the most severely impacted domains.

## Data Availability

The original contributions presented in the study are included in the article/**Supplementary Material**, further inquiries can be directed to the corresponding author.
