# Supplementary figures and images for: Functional status of pediatric patients with trauma and risk factors for mortality from a single center in China
Source: Front Pediatr. 2023 May 3;11:1051759. doi: 10.3389/fped.2023.1051759 (PMC10188922; doi:10.3389/fped.2023.1051759)

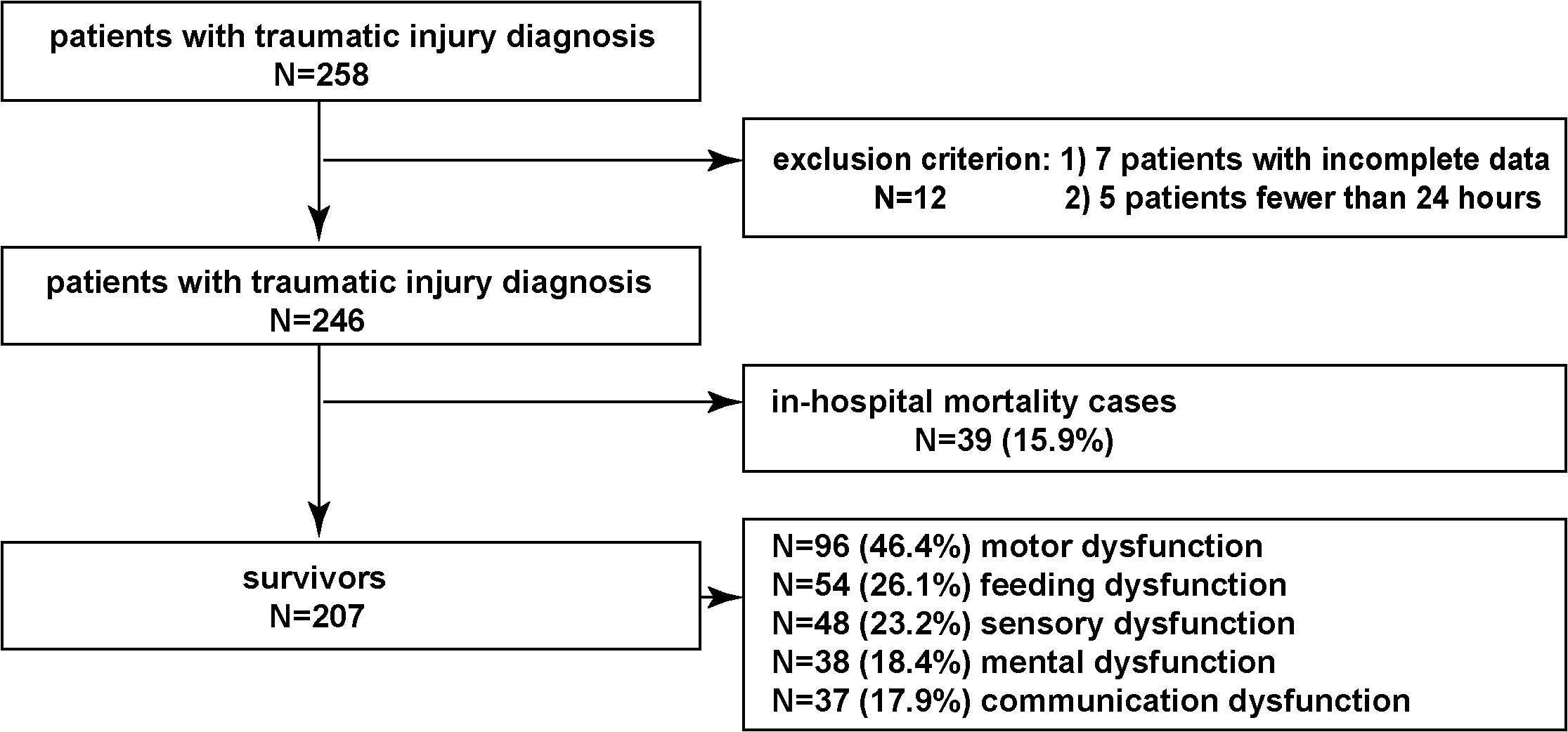

Supplement: Supplementary file 1 [file Image1.tif]
